# Cases of Inhalation of Ether in Labor

**Published:** 1847-10

**Authors:** Walter Channing


ART. III.—*Cases of Inhalation of Ether in Labor.* By WALTER CHANNING, M. D. Second Edition, Boston, published by White and Potter, Chronotype Office, 15 State St., 1847: 12 mo. pp. 44.

The last number of our Journal contained an account of several cases of the Inhalation of Ether in Labor, communicated for the Boston Medical and Surgical Journal by Walter Channing, M. D., Professor of Midwifery in the Mass. Med. College. These cases, together with a few others occurring subsequently to the communication referred to, have been collected and published in a neat pamphlet form, under the above title. Such has been the demand for the publication, owing to the general interest felt in the subject, that the first edition was speedily exhausted, and a second called for. To Prof. Channing is due the merit of being the first in this country to report a series of experimental investigations of the applicability of Ether in Midwifery Practice; and it is fortunate for the subject that it is in the hands of one so competent and judicious. Our commendations of course can add nothing to the high reputation which Prof. C. has long enjoyed as a teacher and practitioner, but it may not be so well known at a distance from the place of his abode, that his philanthropic zeal is not less than his professional ardor. We can well imagine that his interest in this subject has been in no small degree owing to its humane bearings, and we are well assured that to be instrumental in the alleviation of human suffering, would possess for him more charms than that distinction arising from purely scientific researches.

To divest Labor of suffering, or, even, to provide safe and efficient means for its mitigation, is certainly a consummation devoutly to be wished. With the facts as yet before the profession, however, it would be presumptuous to hazard an opinion as to the extent to which these ends are attainable by the inhalation of ether. The observations of Prof. Channing demonstrate that it may be successfully employed in this way, nor have any unpleasant consequences been thus far reported. But, as Dr. C. intimates, we have not facts enough at present to afford any general deductions as to the extent to which it may be safely and properly made available in midwifery. We trust that Dr. C. will continue his investigations, and that others will contribute facts; but, at the same time, we would enjoin upon all who make trial of the Ether under these circumstances, to observe the utmost prudence and care, so as to avoid, if possible, accidents, which may diminish, or prevent its usefulness, by rendering practitioners fearful of employing it when it would be perfectly safe. The preface of Dr. Channing's pamphlet contains remarks so philosophical and pertinent in their relations to the subject, that we make no apology for transferring them to our columns. It also embraces some observations which were made after the publication of the first edition. We copy the preface entire:—

"In publishing another edition of this pamphlet, I beg leave to call the attention of the reader to the cautions which are given in it in regard to the trials of Ether in Labor. As has been said at home and abroad, these trials are to be regarded as "experimental." Time is to determine what is to be the precise place of this agent in midwifery practice. I have heard of trials by different physicians in this city and neighborhood, of Ether in Labor. In one case, in which it was used during the process, it was afterwards inhaled to relieve *after pains*, from which the patient had in former labors suffered more than in the pains of delivery. It was fully used, and was very successful. In four cases in the hands of another physician, its power over pain was very striking. In one more so than in any one I have met with. The patient was a feeble person, and after a previous labor had been in great danger from puerperal fever. This patient was as quiet, as perfectly at rest while *etherized*, and while efficient pains were present, as if she had been in sound sleep. Her child was born without the least consciousness on her part of the fact. She recovered perfectly well. In one of these four cases severe hemorrhage occurred in about an hour after delivery. Its effects were of some hours duration. She had a perfectly good recovery. I have heard of many other instances in which ether has been used, and have very rarely learned of any untoward results. The statements have been of great relief from suffering and entire safety. A friend has used it in two cases, in one solely on account of the exceedingly severe character of former labors. The relief was perfect. What is the precise agency of ether in labor, we know not. The time for philosophy has not come. Every case I have seen has been an *individual*. It has had its own characters. It has presented its own facts for observation and study. It is the duty of medical men to put down in writing every case they see, just as they see it. The temptation is to rest satisfied with the wonder which such facts as etherization give birth to. But physicians have a higher function in this matter. The use of ether is related to the most interesting and important physiological processes in nature, and as such should they be noticed, and investigated.

There is duty, and there is labor, in this use of ether. No man has a proper sense of his responsibleness in this matter, who does not regard it in all its relations. He is using an agent which suspends a law of the animal economy. He uses an agent which separates cause and effect, action and pain, in a process in which this relation has been established by universal observation and experience. A process, labor, which has till this day been attended by severer pain, and longer continued than is observed in any other, has been relieved from suffering and in many cases made positively pleasurable. Is not such a fact of the deepest interest, and should it not arrest every mind whose office it is to observe it? But, once more, the use of ether in labor is still in an important sense experimental; many, many trials are made with it every day, but we wait still for the principles. The generalization which is to establish principle in this as in any other subject of experimental research must come of many facts. Physicians must furnish them. Let caution mark every step of an experiment which has such important issues, and it will be settled, it may be in not many days, what are precisely the rules for the use of ether, both in regard to the cases in which it way be tried, and the best methods of using it.

I understand there has been some disappointment here in regard to the effects of ether in labor. It has not in all trials accomplished what the observations of others have led physicians to expect.—This deserves a distinct place in this early history, and it is most earnestly hoped that such cases will be fully reported.

Would it not be useful to know by whom the ether has been employed in cases of failure, if such have occurred,—exactly how it was used.—what were its principal effects, and what, if any, facts there were in the cases, which might have prevented its ordinary results? Was the full effect produced in these cases? Was the sponge saturated with ether? Was it pure? Was it inhaled until its full influence was experienced by the patient? It has been thought the safest, and best way so to use ether in these and in all cases, as will place the patient fully under its power. After having done this it is perfectly easy to continue the state of *etherization* by very slight inhalations. There is no danger of asphyxia if atmospheric air is properly admitted to the lungs, as through the sponge when that is used, or by the immediate mixture of the air with the ether vapor, as in the inhaler. Has this been the mode in which ether has been used in the cases of its alleged failure, or imperfect action? It is exceedingly important to have such cases most fully reported. Has not the negative evidence as much, nay has it not more value, in so important a matter as this, than has the positive. Is not the demand a reasonable one, which has for its object to obtain reports of these cases,—of all the facts in all cases?

It is occasionally found that patients from doubting the use of ether to prevent pain, from fear of the result, and from other considerations, have refused to have it employed in their cases. This of course is to be expected in this early day of its trial, and is not to be regretted. I am told that there are physicians who absolutely refuse to employ ether in midwifery practice at all. They have no confidence in its powers, and will make no such trials with it as might reveal to them what those powers are, or support them in their opposition to it. This mode of viewing the subject comes of a peculiar moral or intellectual idiosyncrasy which absolutely prevents such men from seeing things as do others, or of wishing to see them by experiment, however easy, and however safe, such trials may be. It resolves into denial where there are no facts to determine the judgment,—into reasoning, without any true use of reason. It is not to be regretted that this fact in the history of etherization exists. It says of it just what has been said of, and to all other discoveries, and frequently the most important, and so allies it to them all. Let those who have tried ether in labor, continue to try it when the circcumstances of cases demand, or authorize its use, and with the caution which such a duty involves ; and that doubt, which, in the instances in which it now exists seems most authoritative, may pass away, and the authority come to be on the side of the remedy of pain.

Shall ether be used in all cases, or do you mean so to use it? are questions which are frequently asked. For myself, I answer no. Cases of labor have come under under my care since this pamphlet was published, in which the process was allowed to proceed without the agency of ether. Two of them were cases of consultation, and were both first labors, and of nearly forty hours continuance. I saw no reason for interfering. One of the cases was rapidly advancing, and the other was aided by ergot. The pains were not so severe as to lead the patients to ask for means which might make them less ; or incline them to use the means when named. The time will very probably come when the question propounded in the beginning of this paragraph will get a different answer. A proper use of our present knowledge authorizes the one given above.

Since writing the above a case has come under my observation of the application of ether, which may not be out of place in this portion of this pamphlet, though not belong to its proper subject.— I give it as an *individual* case. It is a single instance of the use of ether in a disease in which I have not heard that it has been before used.

Miss ——, aged 26, was seized on Sunday, July 4th, 1847, with very severe pain about the root of an incisor tooth, which had been cut off seven years before, and to which a tooth had been attached by a pivot. The pain became so intolerable in the afternoon that she had the tooth removed. This gave no relief. The pain continued through the night and the next day, Monday, July 5th.— Through that whole day the pain continued so that she used up an ounce of laudanum, applying it constantly by means of cotton to the tooth, and to the neighboring parts. At about 11, P. M., the pain suddenly ceased, and she was very soon after seized with vomiting of a most distressing character. The attacks occurred at intervals at from ten to fifteen minutes, and at length had complicated with them severe cramps of the extremities, more especially the hands, which were also numb, and with strong cramps of the abdominal muscles. The suffering was so intense that as many as five persons were required to be with, and aid her. I was called in consultation by the family physician about 9, A. M., July 6th.

There had been a somewhat longer interval from the preceding attack of vomiting when I arrived at the address. But the cramps were very severe, and being so extensive presented a case of great suffering. She vomited while I was present. The effort was violent, but followed only by a little mucus, or a little bitter, and occasionally acid fluid. I prescribed the hydrocyanic acid, and left her, to return after making some medical visits. I did so, and took witn me ether, and a sponge for its inhalation. I found she had vomited the acid about fifteen minutes before I arrived, and was still suffering from cramp. This was about 10 o'clock, eleven hours from the beginning of the disease, during which time she had suffered most severely, and had not slept at all.

I suggested the use of ether. The physician in attendance acceded to the proposal, and it was inhaled. The following was the condition of the patient: Stomach quiet. Cramps and numbness present. Pulse 48. Respiration natural. Complexion and temperature natural. Tongue clean. Head clear. Pressure over region of stomach, and over abdomen generally, gives no pain.— The sponge was well saturated. At first the breathing was embarrassed by the vapor, and slight choking was noticed. This however soon passed off, and she inspired the vapor freely. In about a minute she became perfectly still. Her breathing was hardly perceptible. Her countenance had the expression of perfect repose. The color was as before inhalation. Her pulse was 96. Breathing slow, but sufficiently deep. Temperature as at first. I asked her to open her eyes. An ineffectual attempt was made, the lids scarcely parting. I asked what were her feelings. She made no answer. The sponge was removed, and she remained in this state some minutes. She now roused up. Said "how sick I am! that ether makes me sick." She soon vomited. The effort was very strong, as strong as any previous one, producing much distress, but accompanied by *no cramps;* only a slight *numbness* of the *hands.*— She hardly complained of it. At length the vomiting ceased.— The numbness, and all disagreeable sensations, passed away. She had a threatening of retching once afterwards; but which was only expulsion of flatus. To quiet the stomach, ice in a towel was now applied freely to the epigastrium and left hypochondrium, and with marked comfort. Ice was also urgently demanded to be held in the mouth. It was exceedingly grateful. I left her between 11 and 12 so much relieved that I believed the disease had subsided, and improvement was constantly advancing. The pulse was again 84, and though some paleness was present, and some faintness complained of, I felt she was doing well. I saw her again about 1, P. M., and found good progress had been made. Nausea, cramps and numbness, had entirely left her. She enjoyed the ice greatly, and asked for nourishment. I left her and was to learn if any return of vomiting, oe any other trouble occurred. She expressed her gratitude for the relief she had derived from the use of ether.

July 7th, 10, A. M.—Patient remained comfortable through the day. Restless at night, suffering much from the great heat of the weather and from a rash which has appeared extensively on the skin. It should have been said before that a rash was abundant on Sunday the 4th, and disappeared with the pain in the face, on Monday evening, just before the attack of vomiting and cramps. Was never troubled with this skin disease till since a very severe and dangerous attack of the erysipelas, some months ago. Rash now on face, neck and back. Skin hot. Pulse full, not very frequent. Tongue coated. Thirsty. In short is very much as she before has been during attacks of rash. There has been no trouble of the stomach, no cramps, no numbness, since the speedy relief which followed inhalation of ether yesterday. Expresses more fully than before how much she feels indebted to inhalation.

Ether in this case was used on account of the violence of spasm, cramps, which constituted its chief suffering. Cramp did not happen once after its use."

